# The relationship between physical activity, sedentary time, and cognitive function following bariatric surgery

**DOI:** 10.1016/j.soard.2025.01.010

**Published:** 2025-02-14

**Authors:** Urja Bhatia, Dale Bond, Jeffrey A. Ciesla, John Gunstad, Ian Carroll, Ross Crosby, James E. Mitchell, Christine M. Peat, Kristine Steffen, Leslie Heinberg

**Affiliations:** a Department of Psychological Sciences, Kent State University, Kent, Ohio; b Departments of Surgery and Research, Hartford Hospital/Hartford HealthCare, Hartford, Connecticut; c Department of Nutrition, The University of North Carolina at Chapel Hill, Chapel Hill, North Carolina; d Sanford Research, Sanford Health, Sioux Falls, South Dakota; e Department of Psychiatry and Behavioral Science, University of North Dakota, Fargo, North Dakota; f Department of Psychiatry, The University of North Carolina at Chapel Hill, Chapel Hill, North Carolina; g School of Pharmacy, North Dakota State University, Fargo, North Dakota; h Department of Psychiatry and Psychology, Cleveland Clinic, Cleveland, Ohio

**Keywords:** Cognitive impairment, Obesity, Sex differences, Weight loss surgery

## Abstract

**Background:**

Obesity is associated with cognitive impairment. Metabolic and bariatric surgery (MBS) improves cognitive functioning and weight loss is not a primary mechanism. Physical activity (PA) and sedentary time (ST) may play an important role in cognitive outcomes following MBS, though few studies have examined this possibility.

**Objectives:**

Prospectively examine the relationship among PA, ST, and cognitive function following MBS, as well as possible sex differences in these associations.

**Setting:**

Data were collected at 2 health centers in the United States.

**Methods:**

MBS patients (n = 138, 42.9 α 10.5 years of age, body mass index of 46.3 α 7.3 kg/m^2^) completed the National Institute of Health Toolbox, a computerized neuropsychological battery, and wore an accelerometer for 7 days at preoperative baseline and 1, 6, and 12-month postoperative follow-up to measure ST, light-intensity PA (LPA), and moderate-to-vigorous intensity PA (MVPA).

**Results:**

Total attrition rate was 33.3%. Multilevel modeling showed that ST was negatively associated with List Sorting Working Memory ($\beta_{0j}$ = −.02, *t*(379.77) = −3.89, *p* <.001, 95% *CI* = [−.03, −.01], *d* =0.26) and Picture Sequence Memory ($\beta_{0j}$ = − .02, *t*(337.93) = − .02, *p* = .009, 95% *CI* = [− .03, − .004], *d* = 0.47), and positively associated with Flanker Inhibitory Control and Attention ($\beta_{0j}$ = .02, *t*(378.56) = 2.89, *p* = .004, 95% *CI* = [.005, .025], *d* = 0.22). MVPA was positively associated with Card Sort ($\beta_{0j}$ = .10, *t*(139.02) = 2.79, *p* = .006, 95% *CI* = [.03, .17], *d* = 0.58). No significant relationships were found between LPA and cognitive function, and no sex differences were found in any associations.

**Conclusions:**

The current study links ST and MVPA to cognitive function, though ST was both positively and negatively associated with test performance. Such findings suggest a role for movement and activity levels in cognitive outcomes following MBS, though further investigation is needed to clarify possible mechanisms.

Obesity is associated with adverse health outcomes, including type 2 diabetes (T2D), obstructive sleep apnea, and cardiovascular disease [1]. Previous work also shows that obesity is associated with accelerated cognitive aging [2], and persons with class III obesity (i.e., body mass index [BMI] ≥ 40 kg/m^2^ [3]) appear at particularly high risk for cognitive dysfunction [4]. Though the mechanisms remain unclear, research links low-grade systemic inflammation, insulin resistance, and poor glycemic control prevalent in persons with obesity to cognitive dysfunction [5,6].

Metabolic and bariatric surgery (MBS) is the most effective treatment for class III obesity and produces significant weight loss and reduction or resolution of comorbid conditions [7]. MBS has also been associated with improved cognitive function, with gains found in memory and executive function [8,9]. However, changes in BMI and/or comorbid conditions do not fully account for improvements in cognitive function [8], highlighting a need to identify other contributors.

Greater physical activity (PA) and reduced sedentary time (ST) may be underappreciated contributors to cognitive improvement following MBS, as they are associated with reduced risk of dementia and cognitive decline in other populations [10,11]. To date, much of the literature has focused on the potential cognitive benefits of moderate-to-vigorous intensity PA (MVPA) when examining relationships between PA and cognitive outcomes [12]. Notably, changes in PA following MBS are often minimal and no significant change in device-measured MVPA is found in the first 12 months after surgery [13]. Recent work raises the possibility that in addition to MVPA, other changes in movement – such as reduced ST or increased light-intensity PA (LPA) – might contribute to cognitive improvement following MBS. For example, a recent meta-analysis found increased LPA following MBS [14] and LPA is linked to cognitive benefits in older adults [15]. Similarly, small reductions in ST were maintained 3 years postsurgery [16] and lower ST has also been associated with better cognitive functioning in other populations [17].

In combination, these findings raise the possibility that changes in ST, LPA, and MVPA may be associated with cognitive improvement following MBS. The current study examined this possibility as well as possible sex differences, as our previous work found greater levels of ST were associated with poorer working memory in women prior to MBS [18]. These findings emphasize the need to understand how postoperative changes in activity-related behaviors relate to changes in cognitive function after MBS and the role of sex in this relationship.

The current study examined: 1) changes in cognitive function from presurgery to 12 months postsurgery; 2) whether ST, LPA, and MVPA are associated with cognitive functioning from presurgery to postsurgery, independent of weight loss; and 3) possible sex differences in the associations among ST, LPA, MVPA, and cognitive function over time. We hypothesized that cognitive function would improve in all domains except language [19]. We also hypothesized that greater ST and lower LPA and MVPA would be associated with poorer cognitive functioning, and that these associations would be stronger for women [18].

## Materials and methods

Baseline assessment occurred prior to surgery, and follow-up assessments occurred at 1-month, 6 months, and 12 months postsurgery. Cognitive function, PA, and body composition data were collected at each study visit. The study protocol was approved by the appropriate Institutional Review Boards. Written informed consent was obtained from all participants prior to participation in the study.

### Participants

Data were collected from 145 participants pursuing MBS as part of a larger project [20]. Inclusion criteria for the larger study included 18-65 years of age, in evaluation to undergo either Roux-en-Y gastric bypass (RYGB) or sleeve gastrectomy (SG), and availability for 2 years of follow-up after surgery. Exclusion criteria included tobacco use or alcohol or substance use disorder in the past year; severe psychiatric disorder that may affect the participant’s ability to comply with the study protocol; current routine use of weight loss medication or medications known to significantly influence gastrointestinal transit time; history of gastrointestinal disease or disorder; history of gastrointestinal surgery, not including appendectomy or cholecystectomy; antibiotic, prebiotic, or probiotic use in the past month; positive urine drug screen for nonprescribed medications; pregnancy or breast feeding; inability to participate in PA or dietary monitoring; or any medical condition that would put the individual at risk in the study. The sample for this archival study was further reduced by excluding potential participants with missing actigraphy data at all timepoints (n = 7), leaving 138 participants for primary study analyses.

### Measures

#### Cognitive function

The National Institute of Health (NIH) Toolbox for the Assessment of Neurological and Behavioral Function Cognition Domain is a computerized neuropsychological test battery that is administered via iPad. The NIH Toolbox assesses performance across several cognitive domains and has demonstrated strong validity and reliability [21]. Raw scores were converted into age-corrected standard scores with a mean of 100 and standard deviation of 15, with higher scores representing better performance for each of the following 6 subtests:

#### Dimensional change card sort test.

This test of executive function quantifies set shifting, which is the ability to mentally alternate between different processes/tasks. Participants are shown a picture of an object which vary in shape and color, and asked to select one of two target pictures that matches either the shape or color of the original object, depending on which instruction is given.

#### Flanker inhibitory control and attention test.

This test is a measure of attention in which participants are required to make quick decisions regarding the direction of a stimulus.

#### Picture vocabulary test.

This test is a measure of language that assesses single-word vocabulary knowledge. Participants are given a word aloud, shown four target pictures, and asked to select the picture that matches the meaning of the word.

#### List sorting working memory test.

A measure of working memory, participants are presented with a series of stimuli within the categories of food and/or animals and asked to recite them back in size order from smallest to largest, first for only one category followed by for both categories.

#### Picture sequence memory test.

This test assesses episodic memory and requires participants to learn and recall a sequence of pictures.

#### Pattern comparison processing speed test.

This test is a measure of processing speed in which participants are shown two pictures presented together and required to quickly identify whether the pictures are the same or different.

### Physical activity

PA was assessed using the ActiGraph GT9X Link (AG; Actigraph LLC). The AG records movement, rotation, and body position using a combination of a triaxial accelerometer with a wear time sensor and secondary accelerometer, gyroscope, and magnetometer. Participants were asked to wear the AG on the waist during all waking hours, with the exception of swimming and bathing, for seven consecutive days at all time points. Valid wear time criteria included 4 days (including one weekend day) with at least 600 mins of wear time between the hours of 7 am and 11 pm Insufficient actigraphy wear time was coded as missing PA data at that time point. Variables of interest included average daily minutes spent in ST (<1.5 metabolic equivalents, METs), LPA (i.e., 1.5 to < 3.0 METs), and MVPA (i.e., ≥3 METs) [22].

### BMI and excess body weight loss

Height and weight data were collected at each study visit and used to calculate BMI and percentage of excess body weight loss (%EBWL). To calculate %EBWL, the difference between baseline weight and weight at each time point following surgery was divided by the difference between baseline weight and weight necessary to be at a BMI of 25 kg/m^2^, multiplied by 100.

### Analytic strategy

Descriptive statistics were used to characterize the sample. Prevalence of mild cognitive impairment (MCI), defined as <1.5 SD below the mean on any single cognitive test, were computed [23]. Independent samples *t*-tests and chi-square tests compared participants with and without missing data on demographic variables. Given the use of within persons data, multilevel modeling was utilized in the current study. Multilevel modeling examined changes in cognitive functioning, SB, LPA, MVPA, and %EBWL over the course of the study.

In all models, age-corrected standard scores from each subtest of the NIH Toolbox represented the outcome variable and restricted maximum likelihood approximation was used. A two-level multilevel null model with time (level one) nested within persons (level two) was conducted first with no predictors in the model to calculate the intraclass correlation coefficients (ICCs). ICCs ranged from .4 to .81, confirming the need for a multilevel modeling approach.

Growth models were tested for all cognitive function and activity variables with and without the random effect of time and their −2 log likelihood functions were examined to determine whether to include the random effect of time in the analyses. The difference between the two models was not significant for any of the cognitive function variables, ST or LPA. However, the growth models for MVPA and %EBWL required a random effect of time, therefore final analyses included the random effect of time.

Multilevel modeling was used to determine whether changes in cognitive functioning following MBS are associated with SB, LPA, and MVPA after controlling for % EBWL. Sensitivity analyses were conducted excluding participants with insufficient actigraphy wear time data at any time point, coded as missing, that were retained in the full models. To examine the potential moderating role of sex in these associations, the full models were tested including interaction effects of sex and both ST and LPA were examined. Male was coded as 0, and female was coded as 1.

Given that multilevel modeling is robust to missing data, participants with missing data were retained in the model. The Hochberg step-up procedure was used to correct for multiple comparisons. All analyses were conducted using SPSS version 29.

## Results

Sample characteristics and descriptive statistics for all variables at all study visits are presented in Table 1.

Total attrition rate was 33.3%. No differences were observed in participants with and without missing data for age, sex, or race/ethnicity (all *P* > .05). However, at baseline, participants with missing data (*M* = 47.4, *SD* = 7.4) had significantly higher BMI than participants without missing data (*M* = 43.9, *SD* = 6.4), *t*(136) = − 2.77, *p* = .006. Participants with missing data (*M* = 240.1, *SD* = 70.9) also had significantly fewer average daily minutes of LPA than participants without missing data (*M* = 282.3, *SD* = 92.6), *t*(121) = 2.84, *p* = .005. No significant differences were found between those with and without missing data for SB, MVPA, or any NIH Toolbox subtests (all *P* > .05).

Growth models revealed significant positive change over time for Flanker ($\pi_{1j}$ = .22, *t*(309.28) = 2.34, *p* = .02, 95% *CI* = [.04, .41]), List Sort ($\pi_{1j}$ = .50, *t*(309.89) = 5.28, *p*<.001, 95% *CI* = [.32, .69]), Picture Sequence ($\pi_{1j}$ = .57, *t*(327.19) = 4.17, *p* <.001, 95% *CI* = [.30, .83]), Pattern Comparison ($\pi_{1j}$ = .70, *t*(318.96) = 5.76, *p* <.001, 95% *CI* = [.46, .93]), and Card Sort ($\pi_{1j}$ = .85, *t*(314.51) = 6.37, *p* <.001, 95% *CI* = [.58, 1.11]). Positive change over time thus indicated that scores on each of these subtests increased over the course of the study. At 12-months postsurgery, 20.7% of participants met criteria for MCI compared to 37% at preoperative baseline. No significant change over time was seen for Picture Vocabulary. There was also no significant change over time seen for SB, LPA, or MVPA (all *P* > .05). A significant positive change over time was found for %EBWL ($\pi_{1j}$ = 5.33, *t*(77230.78) = 9.71, *p*<.001, 95% *CI* = [4.26, 6.41]), indicating an increase in excess body weight lost over the course of the study.

Level one predictors were entered as simultaneous predictors of NIH Toolbox subtest scores. An example of this equation is shown below:

$$\mathrm{Flanker}=\beta_{0j}+\beta_{1j}({\mathrm{Time}}_{ij})+\beta_{2j}({\mathrm{SB}}_{ij})+\beta_{3j}({\mathrm{LPA}}_{ij})\;\;+\beta_{4j}({\mathrm{MVPA}}_{ij})+\beta_{5j}(\%{\mathrm{EBWL}}_{ij})+r_{0j}+e_{ij}$$

After controlling for %EBWL, there was a positive relationship between ST and Flanker ($\beta_{0j}$ = .02, *t*(373.35) = 2.91, *p* = .004, 95% *CI* = [.005, .025], *d* = 0.22), suggesting that increases in ST were associated with an improvement in Flanker scores over the course of the study. Negative relationships were found between ST and both List Sort ($\beta_{0j}$ = −..02, *t*(372.67) = −3.88, *p* <.001, 95% *CI* = [−.03, −.01], *d* = 0.26) and Picture Sequence ($\beta_{0j}$ = − .02, *t*(321.94) = − 2.65, *p* = .009, 95% *CI* = [− .03, − .004], *d* = 0.47), suggesting that decreases in ST were associated with better performance on List Sort and Picture Sequence over the course of the study.

A positive relationship was found between MVPA and Card Sort ($\beta_{0j}$ = .10, *t*(139.02) = 2.79, *p* = .006 95% *CI* = [.03, .17], d = 0.58), suggesting that increases in MVPA were associated with an improvement in Card Sort scores over the course of the study.

No significant associations were found between NIH Toolbox subtests and LPA. See Table 2.

Sensitivity analyses revealed that findings did not change on any of the full models when excluding participants with insufficient actigraphy wear time data at one or more time points.

No significant interaction effects were found for sex by ST, sex by LPA, or sex by MVPA for any NIH Toolbox subtests.

## Discussion

The current study examined the association between cognitive function and ST, LPA, and MVPA in MBS patients preoperatively to postoperatively and explored whether these associations differed by sex. Results showed that after controlling for weight loss, ST was both negatively and positively associated with cognitive test performance. Increases in MVPA following MBS were associated with greater set shifting ability. No significant associations were found between LPA and any of the cognitive tests and no significant sex differences emerged. Several aspects of these findings warrant discussion.

Cognitive test performance significantly improved over the course of the study in all domains except language, similar to past studies in MBS patients [8,9,19]. The current study is the largest to date to prospectively assess cognitive function using the NIH Toolbox in MBS patients. A previous study assessing cognitive function at baseline and 2-year follow-up found improvements on the Dimensional Change Card Sort Task and stable performances on all other tests [24]. The current study extends these findings and indicates that acute changes in cognitive function found using other instruments [8,9] can also be detected using NIH Toolbox, including improvements in attention, processing speed, memory, and executive functioning as early as 1 month post-operatively. If confirmed through additional studies that account for possible measurement effects [25], such findings suggest that MBS is consistently associated with cognitive improvements prior to substantial weight loss and encourage further examination to clarify underlying mechanisms.

No changes emerged in ST over the course of the study and cognitive test performance showed mixed associations with ST. Greater ST was associated with poorer episodic and working memory performance, consistent with past work in older adults [17]. However, analyses also showed that greater ST was associated with better attention and inhibitory control performance. The results for ST and cognitive variables ranged from small to moderate, suggesting a small but meaningful effect of ST on cognitive function. The exact explanation for this contradictory pattern is unclear. ST is known to exacerbate many proposed physiological mechanisms for obesity-related cognitive impairment including systemic inflammation and insulin resistance [26], so the current findings linking greater ST to poorer test performance is not surprising. It is less clear how greater ST would also be associated with better cognitive function, but a possible explanation may be found in the specific types of sedentary activities that individuals engage in. Previous studies found that more time spent engaging in sedentary leisure activities with low cognitive demand (e.g., napping, watching TV) are unrelated or even negatively related with cognitive functioning, while more cognitively stimulating (e.g., reading, writing, work-related SB) and social leisure activities (e.g., spending time with others) have been associated with better cognitive functioning and may be protective against adverse cognitive outcomes such as dementia [17,27-29]. Such findings suggest that more fully elucidating the type and nature of sedentary activities and cognitive demands of such activities may be necessary to better understand the relationship between ST and cognitive functioning in MBS and other patient samples.

Neither LPA nor MVPA significantly changed over the course of the study. LPA was unrelated to cognitive test performance in the current study, contrary to previous findings in older adults [15]. This pattern may in part be due to the lack of significant increase in levels of LPA following MBS, as it is possible that there is a threshold of LPA that is necessary to achieve cognitive benefits that the current sample did not reach. This possibility is supported by previous work that has shown that exercise interventions targeting lower intensity activity can improve cognitive function [30] and encourage examination of this possibility in MBS populations. Similarly, changes in ST and MVPA showed significant associations with cognitive function, despite the lack of significant changes in these PA indices. This pattern may suggest a greater sensitivity for these aspects of PA, in that even subtle changes in ST and MVPA, or potentially even maintenance of presurgery levels, may be associated with changes in cognitive functioning following MBS.

Increases in MVPA were associated with better set shifting ability over the course of the study, consistent with previous work in other populations that have shown greater executive functioning performance with greater levels of MVPA [12]. These findings showed a moderate effect and suggest that increasing MVPA following MBS may lead to improvement in executive functioning, though MVPA levels often remain low following MBS and many patients exhibit difficulty maintaining MVPA levels over time [13]. One promising intervention is tailored behavioral counseling to increase moderate intensity walking at home prior to undergoing surgery, which has also been shown to result in greater PA postoperatively [31]. Additional intervention studies are needed to displace SB with LPA and MVPA following MBS to increase variability and examine the potential effects on cognitive functioning.

No significant sex differences emerged in the associations among ST, LPA, MVPA, and cognitive function in the current study. These findings suggest that the greater cognitive benefits found for PA for older adult women compared to men may not extend to MBS populations [32]. However, complicating this interpretation, previous findings from the current sample at baseline found that sex differences were evident preoperatively, in that women with greater levels of ST displayed poorer working memory while greater ST was associated with greater set shifting ability in men [18]. Given that our sample included only a small number of men (n = 27), it is also possible that analyses were underpowered specific to sex differences. Additional studies are needed to clarify possible sex differences in the association between ST/PA and cognitive function in MBS settings.

This prospective study objectively measured participants’ time spent engaging in PA and SB, which helps clarify mixed findings from past studies for postoperative changes [13,16]. However, the current work lacked a control group which precludes causal conclusions. Future studies should recruit a nonsurgical comparison group to clarify the role of PA and ST in cognitive improvement in class III obesity, both in combination with and independent of the effects of MBS. The current sample was predominantly women which may limit opportunity to identify sex differences. Additionally, it is possible that range restriction in both PA and cognitive function variables may have influenced findings, given that MVPA levels were low and NIH subtest scores were largely within the average range at the group level at baseline. Future studies should recruit larger, more diverse samples with a more equal distribution of men are needed to increase variability and adequately power analyses for sex differences that may emerge. Finally, given the utilization of archival data for the current study, participants did not report comorbidities or undergo detailed cardiometabolic workup as part of the current study. Common comorbidities (e.g., T2D, obstructive sleep apnea, cardiovascular disease) are also linked to cognitive function [33,34] and improve following MBS [35,36], therefore it is possible that these indices contributed to the improvement in cognitive function and should be examined in future studies to help clarify the role of PA and ST above and beyond that of the amelioration of comorbidities. However, previous work has shown that comorbidity status (i.e., hypertension, sleep apnea, T2D) do not consistently predict cognitive changes 2 years after MBS [37] suggesting that amelioration of comorbidities following MBS may not fully explain cognitive improvement. Similarly, sleep quality is associated with both PA and cognitive function [38] and are related to daytime behavior and activity levels (e.g., napping) [39]. Future studies should objectively assess sleep quality using polysomnography or wrist actigraphy, as well as measure the types of sedentary activities that individuals are engaging in during the day (e.g., napping versus reading) to help clarify mixed findings. Lastly, participants in the current study were 18-65 years of age, which precludes examination of the relationship between PA and cognitive function in older adults. Given that previous work has shown associations between activity levels and cognitive function in older adults [15,17] and the number of older adults pursuing MBS is likely to increase [40], future studies should focus on examining PA and cognitive outcomes in older adults undergoing MBS.

## Conclusions

Mixed findings emerged for the relationship between ST and cognitive function in MBS patients, with greater ST being associated with poorer performance on tests of episodic and working memory and better performance on inhibitory control and attention. A positive relationship emerged between MVPA and set shifting ability, though no relationship between LPA and cognitive function emerged. Future intervention studies are needed to reduce ST and increase PA following MBS to better understand their association with cognitive functioning and promote clinical outcomes. Larger samples are also needed to replicate findings and identify any possible sex differences that may exist.

## Figures and Tables

**Table 1:** Demographics, cognitive function scores, and physical activity indices at all study visits

	Baseline		1-mo		6-mo		12-mo	
	N	M(*SD*) or %	N	M(*SD*)	N	M(*SD*)	N	M(*SD*)
Age, years	138	42.9 (10.5)						
Caucasian	138	73.2%						
Female	138	80.4%						
Not Hispanic/Latino	138	92.8%						
BMI, kg/ m^2^	138	46.2 (7.3)	121	40.5 (6.4)	114	34.3 (6.1)	110	32.6 (6.4)
EBWL			121	27.4 (9.0)	114	58.5 (16.4)	110	66.7 (21.3)
NIH Toolbox Scores								
Flanker	138	97.3 (17.9)	112	99.5 (17.4)	98	97.6 (16.2)	92	101.3 (17.8)
List Sort	138	95.3 (17.3)	112	97.9 (18.2)	98	99.6 (19.2)	92	99.8 (17.2)
Pic Sequence	138	104.7 (16.3)	112	110.9 (16.8)	98	112.1 (17.2)	92	112.6 (17)
Pattern Comparison	138	101.8 (17.2)	111	112.2 (19.3)	98	113.9 (19.8)	92	116.5 (19.2)
Card Sort	138	101.8 (20.2)	111	107.7 (21.5)	98	109.1 (19.1)	92	113.5 (19.9)
Pic Vocab	138	102.2 (13.4)	112	102.5 (11.9)	98	102.8 (11.1)	92	102.4 (12.1)
Physical activity								
ST	123	639.4 (173.6)	105	649.8 (159.8)	97	627.1 (148)	100	651.9 (153.1)
LPA	123	255.9 (81.9)	105	244.8 (72.5)	97	265.2 (89.7)	100	258.2 (83.5)
MVPA	123	15.3 (16.8)	105	16.4 (19.3)	97	22.0 (36.4)	100	19.3 (38.8)

All NIH Toolbox scores display age-corrected standard scores; BMI = body mass index; Card Sort = Dimensional Change Card Sort Test; EBWL = percentage of excess body weight loss; Flanker = Flanker Inhibitory Control and Attention Test; List Sort = List Sorting Working Memory Test; LPA = average daily minutes spent in light-intensity physical activity; MVPA = average daily minutes spend in moderate-to-vigorous intensity physical activity; NIH Toolbox = National Institute of Health Toolbox for the Assessment of Neurological and Behavioral Function Cognition Domain; Pattern Comparison = Pattern Comparison Processing Speed Test; Pic Sequence = Picture Sequence Memory Test; Pic Vocabulary = Picture Vocabulary Test; ST = average daily minutes spent in sedentary time.

**Table 2:** Adjusted and unadjusted associations among sedentary time, physical activity, and cognitive function

Outcome and predictor	Unadjusted					Adjusted				
	β	*t*	*p*	Lower limit	Upper limit	β	*t*	*p*	Lowerlimit	Upper limit
Flanker										
Intercept	87.31	18.62	<.001	78.09	96.52	87.27	18.59	<.001	78.04	96.5
Time	.19	1.84	.07	−.01	.39	.18	.93	.35	−.20	.55
ST	.02	2.92	.004*	.01	.03	.02	2.91	.004*	.01	.03
LPA	.002	.21	.84	−.02	.02	.002	.22	.83	−.02	.02
MVPA	.003	.10	.92	−.05	.06	.003	.09	.93	−.05	.06
EBWL						.002	.06	.95	−.06	.06
List Sort										
Intercept	113.13	23.86	<.001	103.81	122.45	113.13	23.84	<.001	103.8	122.46
Time	.43	4.18	<.001	.23	.63	−.005	−.03	.98	−.38	.37
ST	−.02	−3.66	<.001**	−.03	−.009	−.02	−3.88	<.001**	−.03	−.01
LPA	−.02	−1.84	.07	−.04	.001	−.02	−1.85	.07	−.04	.001
MVPA	.02	.79	.43	−.03	.08	.01	.37	.71	−.04	.07
EBWL						.08	2.69	.008	.02	.15
Pic Sequence										
Intercept	116.4	21.91	<.001	105.94	126.85	115.79	21.77	<.001	105.33	126.23
Time	.62	4.22	<.001	.33	.91	.26	.99	.32	−.26	.77
ST	−.02	−2.58	.01	−.03	−.004	−.02	−2.65	.009*	−.03	−.004
LPA	−.003	−.25	.80	−.03	.02	−.002	−.20	.85	−.03	.02
MVPA	.02	.68	.50	−.04	.09	.02	.48	.63	−.05	.08
EBWL						.07	1.67	.10	−.01	.15
Pattern Comp										
Intercept	105.41	18.65	<.001	94.3	116.52	104.84	18.67	<.001	93.80	115.89
Time	.75	5.72	<.001	.49	1.01	.26	1.08	.28	−.21	.73
ST	.01	1.13	.26	−.01	.02	.01	.97	.33	−.01	.02
LPA	−.01	−.41	.68	−.03	.02	−.004	−.33	.74	−.03	.02
MVPA	−.02	−.57	.57	−.09	.05	−.03	−.91	.37	−.10	.04
EBWL						.10	2.43	.02	.02	.17
Card Sort										
Intercept	104.25	17.0	<.001	92.19	116.31	103.74	16.93	<.001	91.69	115.8
Time	.72	5.04	<.001	.44	1.0	.31	1.20	.22	−.20	.83
ST	.0005	.07	.94	−.01	.01	.0002	−.03	.97	−.01	.01
LPA	−.01	−.78	.44	−.04	.01	−.01	−.72	.47	−.04	.02
MVPA	.12	3.05	.003*	.04	.18	.10	−.79	.006*	.03	.17
EBWL						.08	1.87	.06	−.004	.16
Pic Vocab										
Intercept	102.2	31.51	<.001	95.82	108.58	102.17	31.47	<.001	95.78	108.55
Time	.01	.17	.87	−.12	.14	−.03	−.22	.83	−.27	.22
ST	.001	.28	.78	−.01	.01	.001	.26	.80	−.01	.01
LPA	−.003	−.50	.62	−.02	.01	−.003	−.48	.63	−.02	.01
MVPA	.02	1.15	.25	−.015	.06	.02	1.08	.28	−.02	.06
EBWL						.01	.37	.71	−.03	.05

Hochberg step-up correction was used for all analyses; All cognitive function measures adjusted for age; Adjusted models control for percentage of excess body weight loss; Lower and upper limits indicate 95% confidence intervals; Card Sort = Dimensional Change Card Sort Test; EBWL = percentage of excess body weight loss; Flanker = Flanker Inhibitory Control and Attention Test; List Sort = List Sorting Working Memory Test; LPA = average daily minutes spent in light-intensity physical activity; MVPA = moderate-to-vigorous intensity physical activity; Pattern Com*p* = Pattern Comparison Processing Speed Test; Pic Sequence = Picture Sequence Memory Test; Pic Vocab = Picture Vocabulary Test; ST = average daily minutes spent in sedentary time.

* *P* < .05.

** *P* < .001.
